# Supplementary material for: Molecular Studies of TCF4 Gene and Correlation with Late-Onset Fuchs Endothelial Corneal Dystrophy in the Greek Population: A Novel Cost-Effective Diagnostic Algorithm
Source: Int J Mol Sci. 2025 Nov 24;26(23):11356. doi: 10.3390/ijms262311356 (PMC12692371; doi:10.3390/ijms262311356)
Supplement: Supplementary file 1 [file ijms-26-11356-s001.zip › ijms-3942750-supplementary.pdf]

Supplementary Figure S1. DNA Sequencing result for the F24 sample with a single result in both STR- and TP-PCRs. The arrow indicates the first CTG triplet, another ten triplets are seen in a homozygous state (11/11 normal homozygous sample). Flanking sequences are as expected for borders of the expansion CTG area in the *TCF4* gene.

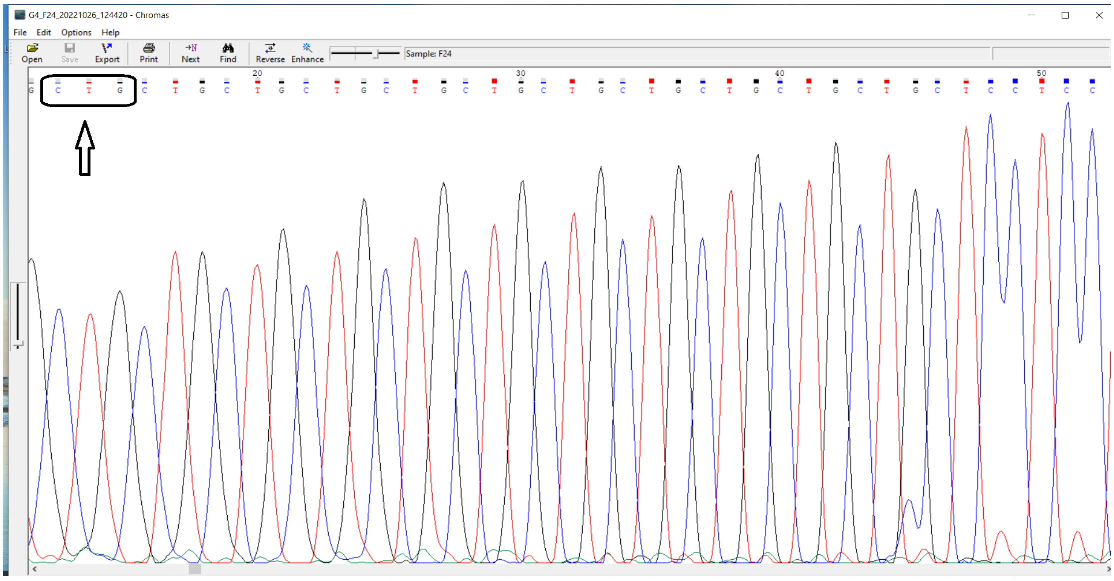

Supplementary Table S1. A comprehensive table to be used as a tool for easy identification of the triplet number and the expected size of STR- and TP-PCR peaks in fragment analysis.

# of STR-PCR Triplets	STR-PCR size (bp)	# of TP-PCR Triplets	TP-PCR size (bp)
5	132	5	69
6	135	6	72
7	138	7	75
8	141	8	78
9	144	9	81
10	147	10	84
11	150	11	87
12	153	12	90
13	156	13	93
14	159	14	96
15	161	15	99
16	164	16	102
17	167	17	105
18	170	18	108
19	173	19	111
20	176	20	114
21	179	21	117
22	181	22	120
23	184	23	123
24	187	24	126
25	190	25	129
26	193	26	132
27	196	27	135
28	199	28	138
29	202	29	141
30	205	30	144

31	208	31	147
32	211	32	150
33	214	33	153
34	217	34	156
35	220	35	159
36	223	36	162
37	226	37	165
38	229	38	168
39	232	39	171
40	235	40	174
41	238	41	177
42	241	42	180
43	244	43	183
44	247	44	186
45	250	45	189
46	253	46	192
47	256	47	195
48	259	48	198
49	262	49	201
50	265	50	204
51	268	51	207
52	271	52	210
53	274	53	213
54	277	54	216
55	280	55	219
56	283	56	222
57	286	57	225
58	289	58	228
59	292	59	231
60	295	60	234
61	298	61	237
62	301	62	240
63	304	63	243
64	307	64	246
65	310	65	249
66	313	66	252
67	316	67	255
68	319	68	258
69	322	69	261
70	325	70	264
71	328	71	267
72	331	72	270
73	334	73	273
74	337	74	276
75	340	75	279
76	343	76	282
77	346	77	285
78	349	78	288
79	352	79	291
80	355	80	294
81	358	81	297
82	361	82	300

83	364	83	303
84	367	84	306
85	370	85	309
86	373	86	312
87	376	87	315
88	379	88	318
89	382	89	321
90	385	90	324
91	388	91	327
92	391	92	330
93	394	93	333
94	397	94	336
95	400	95	339
96	403	96	342
97	406	97	345
98	409	98	348
99	412	99	351
100	415	100	354
101	418	101	357
102	421	102	360
103	424	103	363
104	427	104	366
105	430	105	369
106	433	106	372
107	436	107	375
108	439		
109	442		
110	445		
111	448		
